# The Relationship Between Emotional Eating Behavior and Internet Addiction in Junior High School Students: A Cross-Sectional Study

**DOI:** 10.3390/nu18050800

**Published:** 2026-02-28

**Authors:** Xinru Li, Benli Xue, Haoran Wu, Anfei Luo, Lingli Yang, Xinyi Xu, Zhaodi Chen, Huang Lin, Chichen Zhang

**Affiliations:** 1 School of Public Health, Southern Medical University, Guangzhou 510515, China; 2 School of Health Management, Southern Medical University, Guangzhou 510515, China; 3 Key Laboratory of Philosophy and Social Sciences of Colleges and Universities in Guangdong Province for Collaborative Innovation of Health Management Policy and Precision Health Service, Guangzhou 510515, China; 4 School of Nursing, Southern Medical University, Guangzhou 510515, China; 5 Shenzhen Longhua District Maternity and Child Healthcare Hospital, Shenzhen 518000, China; 6 Southern Medical University Center for Health Policy and Governance (Guangdong Provincial Social Science Research Base), Guangzhou 510515, China

**Keywords:** emotional eating behavior, internet addiction, sleep quality, depression

## Abstract

Objectives: With the rapid development of digital technology, the risk of internet addiction among adolescents has increased. However, the influence mechanism of emotional eating behavior on internet addiction remains unclear. This study aimed to explore the association pathway of emotional eating on internet addiction in junior high school students and test the chain-mediating effects of sleep quality (sleep quality was measured using the PSQI, with higher scores indicating poorer sleep quality) and depression. Methods: Based on data from 3245 junior high school students in Shenzhen, China, internet addiction was measured using Young’s questionnaire, and emotional eating was assessed via the Dutch Eating Behavior Questionnaire’s subscale. The PROCESS macro (Model 6) was used to test the chain-mediating effects. Results: Emotional eating was positively but modestly associated with internet addiction (*β* = 0.024, *p* < 0.01). Three significant mediating pathways were identified: (1) emotional eating → sleep quality → internet addiction (*β* = 0.0062, 14.52% of total effect); (2) emotional eating → depression → internet addiction (*β* = 0.0084, 19.67%); and (3) emotional eating → sleep quality → depression → internet addiction (*β* = 0.0041, 9.60%). Conclusions: Based on cross-sectional data, this study found that emotional eating is associated with internet addiction through the independent and chain-mediating effects of sleep quality and depression, revealing a statistical mediation pathway of “maladaptive emotion regulation → circadian disruption → psychopathology → addictive behavior.” These findings provide a basis for interventions targeting sleep management and emotional regulation.

## 1. Introduction

The rapid advancement of digital technology and artificial intelligence has not only facilitated convenient lifestyles but also enabled students to access abundant knowledge resources effortlessly through the internet. However, for junior high school students navigating the dual critical transitions of adolescence and academic progression, pressures such as academic demands and interpersonal conflicts may easily overwhelm their fragile psychological defenses. This vulnerability is associated with immersion in the virtual world as a means of escaping reality or seeking stimulation, which is linked to an uncontrollable dependence on the internet—a phenomenon termed internet addiction [1]. Defined as a behavioral disorder characterized by prolonged and excessive internet use that individuals struggle to disengage from [2], internet addiction has been demonstrated to be associated with adverse outcomes in adolescents, including academic decline, heightened suicidal ideation [3], increased aggressive behaviors [4], and neurocognitive impairments.

While numerous studies have explored factors influencing internet addiction among junior high school students, a critical research gap persists regarding the impact of emotional eating—a disordered eating pattern—on internet addiction. Emotional eating, a key manifestation of disordered eating, refers to maladaptive eating behaviors triggered by negative emotions [5], characterized by uncontrolled food intake frequency and quantity [6], often involving excessive consumption of high-sugar or high-fat “comfort foods” to alleviate distress [7]. This behavior not only risks binge eating but also is linked to obesity, hyperglycemia, and sleep quality deterioration. From a psychological perspective, emotional eating may be associated with higher vulnerability to internet addiction through an emotion regulation compensation mechanism: when attempts to alleviate negative emotions via eating fail, individuals may turn to the internet for alternative gratification. Empirical evidence supports this link, showing that students with disordered eating exhibit significantly higher tendencies toward internet overuse [8], while obese children and adolescents demonstrate markedly higher prevalence rates of internet addiction compared to healthy peers [9].

Sleep quality and emotional well-being are critical safeguards for junior high school students navigating adolescence. Research confirms that sleep deficits and insomnia exacerbate emotional eating, particularly in response to anxiety and anger [10]. Such eating patterns are further linked to heightened depressive symptoms [11], establishing close interplay among these factors. However, existing studies have only explored the separate associations among emotional eating, sleep quality (PSQI scores, with higher scores indicating worse sleep quality), depression and internet addiction. Notably, consistent with published literature documenting robust reciprocal associations between sleep quality and internet use, as well as between depressive symptoms and internet use, we acknowledge that alternative temporal sequences and bidirectional orderings between these variables are biologically and statistically plausible. Meanwhile, no research has clarified the chained mediating mechanism of sleep quality and depression in the relationship between emotional eating and internet addiction among junior high school students, leaving the specific association pathway yet to be elucidated.

Therefore, the present study aims to examine the chained mediating roles of sleep quality and depression in the association between emotional eating and internet addiction among Chinese junior high school students. Building on this evidence, this study proposes a chain-mediation model—emotional eating → sleep quality → depression → internet addiction. To our knowledge, few studies have investigated this integrated psychopathological mechanism in junior high school populations. By elucidating the underlying pathways through which emotional eating is associated with internet addiction, this research aims to provide novel insights and multi-layered theoretical foundations for designing targeted interventions focused on sleep management and emotional regulation.

### 1.1. Direct Effect of Emotional Eating Behavior on Internet Addiction

Giráldez et al. found a significant positive correlation between emotional eating and excessive internet use in their exploration of emotional eating, internet overuse, and alcohol consumption among college students [12]. Ali’s structural equation modeling analysis of the association between social media addiction and eating behaviors in adolescents and young adults confirmed that social media addiction is directly or indirectly associated with higher levels of emotional eating [13]. Elkin’s investigation of the relationship between emotional eating disorders and problematic internet use in college students revealed positive associations among emotional eating, mukbang addiction, and problematic internet use, with emotional eating also significantly correlated with mukbang addiction [14]. Given the consistent evidence linking emotional eating behavior to social media engagement—a core component of internet addiction—this study proposes the following hypothesis.

**Hypothesis** **1.** *Higher frequency of emotional eating behavior in junior high school students is associated with greater dependency on the internet.*

### 1.2. Mediating Role of Sleep Quality

Emotional eating often coincides with binge eating, which frequently occurs at night. The intense digestive processes triggered by overeating may be associated with sleep onset and poorer sleep quality [15]. A study reported that more severe insomnia symptoms were associated with more frequent binge-eating episodes over a three-month period [16]. Existing research indicates that poor sleep quality and internet addiction are both positively correlated with disordered eating behaviors [17]. Specifically, a weak positive correlation was observed between internet addiction and disordered eating behaviors, while a moderate positive correlation existed between poor sleep quality and such behaviors, with both serving as significant associated with disordered eating in adolescents. Students with sleep problems tend to spend excessive time watching television or browsing social media [18]. In addition, diminished sleep quality is associated with the development of internet addiction [19]. Based on this evidence, this study proposes the following hypothesis.

**Hypothesis** **2.** *Sleep quality mediates the relationship between emotional eating behavior and internet addiction.*

### 1.3. Mediating Role of Depression

Depression has been identified to be associated with both emotional eating and internet addiction [12]. The underlying mechanism may involve adipocytokines (e.g., leptin) secreted by adipose tissue in obese individuals, which induce chronic systemic inflammation and metabolic dysregulation through the release of pro-inflammatory cytokines and free radicals [20,21]. This pathological process may be linked to brain damage via the “inflammation-metabolic dysfunction” pathway, manifesting as neuroinflammation and neurodegeneration, further associated with the onset and progression of depressive symptoms through interactions within the “neuro-immuno-endocrine network” [21]. Zhou et al.’s study on the bidirectional relationship between depression and smartphone addiction in Chinese adolescents demonstrated that depression significantly associated with smartphone addiction, whereas the reverse predictive relationship was unsupported [22]. Furthermore, individuals with depression tend to use the internet more frequently [23] and exhibit a higher likelihood of internet addiction [24]. Negative emotions, such as anxiety, depression, tension, and anger, may drive emotional eating. However, the measurement scale for emotional eating behavior used in this study specifically excludes the depression dimension (see Section 2.2). This design ensures methodological rigor in examining the pathway through which emotional eating influences internet addiction via depressive symptoms. Accordingly, this study proposes the following hypothesis.

**Hypothesis** **3.** *Depression mediates the relationship between emotional eating behavior and internet addiction.*

### 1.4. Chain-Mediating Role of Sleep Quality and Depression

Research indicates that emotional eating correlates with elevated depression and sleep disturbances, and individuals with disordered eating often experience poor or insufficient sleep quality [25]. Adolescents with poor sleep quality are more prone to intense anger, risk-taking behaviors, and depressive symptoms, severely compromising their physical and mental health [26]. A study on depression and internet use among Chinese adolescents confirmed the mediating role of sleep in the relationship between depression and internet use [27]. Huang et al.’s investigation of Taiwanese college students found that depression fully mediated the association between sleep quality and internet addiction [28]. Additionally, brain activity related to emotional regulation is comprehensively associated with circadian rhythms [29,30], and depression may interact bidirectionally with disordered eating and sleep disturbances [31]. Building on this evidence, this study proposes the following hypothesis.

**Hypothesis** **4.** *Sleep quality and depression sequentially mediate the relationship between emotional eating behavior and internet addiction, forming a significant chain mediation pathway: emotional eating behavior → sleep quality → depression → internet addiction.*

## 2. Materials and Methods

### 2.1. Data Sources

This was a cross-sectional study. The survey was conducted from March to April 2024 using a multistage random sampling method. First, one administrative district (LH District) in Shenzhen, China, was randomly selected. Within this district, two nine-year consistent schools were then randomly selected from each of its six subdistricts. Subsequently, 2–4 classes were randomly chosen from Grades 7 to 9 in each selected school, and all students meeting the inclusion criteria within these classes were included as participants. Homeroom teachers provided guidance, counseling, and advocacy services for the students. Before the start of the study, all participating homeroom teachers and students were fully informed of the research purpose, confidentiality protocol, and principle of voluntary participation. During the survey, homeroom teachers instructed students to answer the questions one by one in class. Within the scope of funding feasibility, the study actually distributed 4000 questionnaires, recovered 3751 valid questionnaires, with a questionnaire recovery rate of 93.78%. After excluding 506 individuals with missing key variables, a total of 3245 participants were finally included in the analysis. Since the respondents were under 16 years old, informed consent was obtained from the students and their parents or guardians prior to their participation. All data were collected anonymously and handled with strict confidentiality. The research protocol was reviewed and approved by the Medical Ethics Committee of Longhua District Maternity and Child Health Hospital, Shenzhen (Approval No.: SRE-PCFR/2023024; Approval Date: 14 November 2023) and conducted in accordance with the principles proposed in the Declaration of Helsinki.

### 2.2. Measurements

#### 2.2.1. Dependent Variables

Internet addiction was assessed using Young’s Internet Addiction Diagnostic Questionnaire [32]. This 8-item instrument evaluates: (1) preoccupation with the internet (e.g., persistent thoughts about online activities after disconnection); (2) escalating internet use to achieve satisfaction; (3) difficulty controlling or reducing usage; (4) irritability or dysphoria when attempting to reduce use; (5) frequent exceeding of planned online time; (6) adverse impacts on academics, relationships, or daily life; (7) concealment of internet use from family or friends; and (8) using the internet to escape problems or alleviate negative emotions (e.g., helplessness, anxiety). Items are scored dichotomously (1 = yes, 0 = no), with total scores ranging from 0 to 8; higher scores indicate stronger addiction tendencies. The scale has been validated for Chinese adolescents [33]. In this study, Cronbach’s α was 0.854, KMO = 0.911, and Bartlett’s test of sphericity was significant (χ^2^ = 8569.633, *p* < 0.001), confirming robust internal consistency and structural validity.

#### 2.2.2. Independent Variables

Emotional eating behavior was measured using the Emotional Eating subscale of the Dutch Eating Behavior Questionnaire for Children [34]. This 7-item subscale assesses the urge to eat in response to negative emotions (e.g., “feeling discouraged,” “lonely,” “anxious,” “unsettled,” “fearful,” or “sad”) using a 3-point Likert scale (1 = no, 2 = sometimes, 3 = yes). Total scores range from 7 to 21, with higher scores indicating more severe emotional eating. The scale has demonstrated good validity in Chinese adolescents [35]. Here, Cronbach’s α was 0.945, KMO = 0.926, and Bartlett’s test was significant (χ^2^ = 20,535.932, *p* < 0.001), confirming excellent reliability and validity.

#### 2.2.3. Mediating Variable

Sleep quality was evaluated using the Pittsburgh Sleep Quality Index (PSQI) [36]. The Chinese version employed was translated and validated by Liu [37], demonstrating good reliability and validity in Chinese populations, including adolescents [38,39]. The 18-item scale assesses seven domains (subjective sleep quality, sleep latency, sleep duration, sleep efficiency, sleep disturbances, use of sleep medication, and daytime dysfunction) over the past month. Each domain is scored 0–3, with total scores ranging from 0 to 21; higher PSQI scores indicate worse sleep quality. Cronbach’s α was 0.839, KMO = 0.894, and Bartlett’s test was significant (χ^2^ = 11,419.397, *p* < 0.001), confirming adequate reliability and validity.

Depression was measured using the depression subscale of the Chinese version of the Depression, Anxiety, and Stress Scale-21 (DASS-21) revised by Gong [40]. This 7-item subscale evaluates depressive symptom severity (e.g., low mood, self-deprecation) using a 4-point Likert scale (0 = never, 3 = always). Total scores range from 0 to 21, with higher scores indicating more severe depression. The scale has been validated for adolescents [41]. Here, Cronbach’s α was 0.902, KMO = 0.911, and Bartlett’s test was significant (χ^2^ = 12,803.389, *p* < 0.001), demonstrating strong reliability and validity.

#### 2.2.4. Control Variables

Based on prior studies [42,43], demographic and family-related variables were included as controls: age, sex, household registration type, ethnicity, residence (urban/rural), only-child status, parental relationship status, and parental education levels (father’s and mother’s).

#### 2.2.5. Analysis Strategies

Data were analyzed using SPSS 27.0 and Stata 16.0. Cronbach’s α and Bartlett’s test assessed scale reliability and validity. Normality tests indicated non-normal distributions for key variables; thus, Spearman’s correlation analysis was used to examine variable associations. Harman’s single-factor test evaluated common method bias. Multicollinearity diagnostics for core variables were conducted using Stata 16.0, with variance inflation factor (VIF) values < 10 as the criterion for excluding severe multicollinearity. Chain mediation effects were tested using Model 6 in Hayes’ PROCESS macro. Bootstrap sampling (5000 iterations) estimated standard errors and 95% confidence intervals (CIs) for regression coefficients; effects were deemed significant if CIs excluded zero. Sensitivity analyses were performed using Stata 16.0 to verify the robustness of the core findings.

## 3. Results

### 3.1. Descriptive Statistics

The final sample comprised 3245 junior high school students (1734 males, 1511 females), with a median age of 13.90 years (interquartile range: 13.20–14.60). As the dependent variable violated normality assumptions, Mann–Whitney U and Kruskal–Wallis H tests were employed to analyze differences in internet addiction across demographic characteristics (Table 1). Internet addiction exhibited significant differences by gender (*Z* = −2.546, *p* = 0.011) and parental relationship status (*H* = 118.924, *p* < 0.001). However, no significant differences were observed for household registration type (*Z* = −1.427, *p* = 0.153), residence (urban/rural; *H* = 1.387, *p* = 0.500), ethnicity (*Z* = −1.909, *p* = 0.056), only-child status (*Z* = −0.883, *p* = 0.377), paternal education level (*H* = 5.394, *p* = 0.612), or maternal education level (*H* = 13.639, *p* = 0.058).

### 3.2. Common Method Bias Test

Potential common method bias was examined using Harman’s single-factor test. The results revealed seven factors with eigenvalues greater than 1, with the first factor explaining 22.769% of the total variance—below the empirical threshold of 40%. According to this method’s criteria, no significant common method bias was detected in the study data.

### 3.3. Correlation Analysis of Primary Variables

Following normality tests, variables were found to be non-normally distributed; thus, Spearman’s rank correlation tests were employed to examine the associations between variables. As presented in Table 2, significant pairwise correlations existed among all key variables. Specifically, emotional eating behavior showed a small but statistically significant positive correlation with internet addiction (*r* = 0.066, *p* < 0.01), indicating that greater emotional eating behavior was associated with higher likelihood of internet addiction, albeit with a weak magnitude of effect. Emotional eating behavior also showed a small but statistically significant positive correlation with the Pittsburgh Sleep Quality Index (PSQI; *r* = 0.092, *p* < 0.01), suggesting that more pronounced emotional eating in this population was linked to higher PSQI scores (indicating worse sleep quality), with a weak magnitude of effect. Additionally, emotional eating behavior showed a small but statistically significant positive correlation with depressive symptoms (*r* = 0.113, *p* < 0.01), meaning that higher levels of emotional eating were associated with more severe depressive symptoms, with a weak magnitude of effect. The PSQI was positively correlated with internet addiction (*r* = 0.342, *p* < 0.01), implying that higher PSQI scores (indicating worse sleep quality) were related to a higher probability of internet addiction among these students. Depressive symptoms were positively correlated with internet addiction (*r* = 0.365, *p* < 0.01), indicating that more severe depressive symptoms were associated with a higher likelihood of internet addiction. Finally, the PSQI score was positively correlated with depressive symptoms (*r =* 0.510, *p* < 0.01), suggesting that higher PSQI scores (indicating worse sleep quality) were accompanied by more evident depressive symptoms in junior high school students. Given this relatively strong correlation between the two mediating variables, we conducted multicollinearity diagnostics using variance inflation factor (VIF) tests. The results showed that the VIF values of all core variables were below the conventional critical threshold of 10 (range: 1.01–1.35, mean VIF = 1.24), indicating no severe multicollinearity in the regression model. However, the moderate-to-strong correlation between the two mediators warrants cautious interpretation of the results of the serial mediation model.

### 3.4. Chain Mediation Effect Analysis

**Figure 1:**
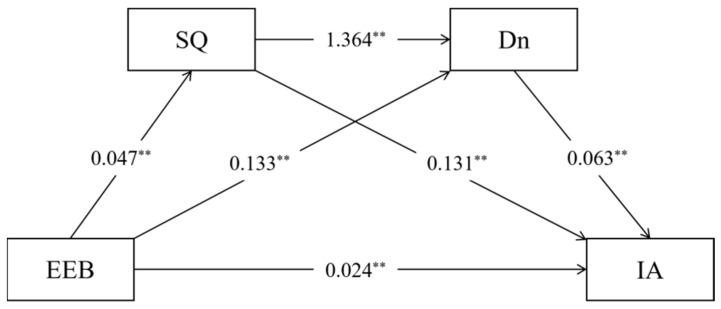
Chain Mediation Model. Note: ** *p* < 0.01; EEB = Emotional Eating Behavior, SQ = Sleep Quality, Dn = Depression, IA = Internet Addiction. SQ was measured by PSQI, with higher PSQI scores indicating worse sleep quality.

We utilized PROCESS 4.2 (Model 6) to analyze the chain-mediating effects of sleep quality and depressive state. We included age, gender, household registration type, residence type, ethnicity, only-child status, paternal education level, maternal education level, and parental relationship as control variables in the model. A resampling procedure with 5000 iterations was conducted, and the Bootstrap 95% confidence interval (CI) was calculated. Figure 1 displays all path coefficients of the chain-mediating model, with all paths demonstrating statistical significance. Overall, emotional eating behavior exhibited a positive association with internet addiction (*β* = 0.024, *p* < 0.01). Both the sleep quality index (PSQI scores, with higher scores indicating worse sleep quality) (*β* = 0.131, *p* < 0.01) and depressive state (*β* = 0.063, *p* < 0.01) also showed positive associations with internet addiction. Within the chain-mediating model, emotional eating behavior had positive associations with the sleep quality index (PSQI scores, with higher scores indicating worse sleep quality) (*β* = 0.047, *p* < 0.01) and depressive state (*β* = 0.133, *p* < 0.01), while the sleep quality index significantly associated with depressive state (*β* = 1.364, *p* < 0.01).

Table 3 presents the total effects, direct effects, and indirect effects of different paths in the chain-mediating model. The direct effect of emotional eating behavior on internet addiction was significant (Bootstrap 95% CI excluding 0), with an effect size of 0.0240, accounting for 56.21% of the total effect. The total indirect effect through sleep quality and depression was also significant (Bootstrap 95% CI excluding 0), with an effect size of 0.0187, accounting for 43.79% of the total effect. Three specific pathways were identified through which emotional eating behavior influenced internet addiction: ① emotional eating behavior → sleep quality → internet addiction (*β* = 0.0062, 14.52% of total effect); ② emotional eating behavior → depressive state → internet addiction (*β* = 0.0084, 19.67% of total effect); ③ emotional eating behavior → sleep quality → depressive state → internet addiction (*β* = 0.0041, 9.60% of total effect).

### 3.5. Sensitivity Analyses

#### 3.5.1. Controlling for School-Level Cluster Effects

Given that this study adopted a multistage random sampling method, a total of 12 schools were included in the final analysis, with a valid sample of 3245 junior high school students and an average cluster size of approximately 270 students per school. The data may have cluster effects at the school level. To address this issue, we included cluster-robust standard errors at the school level in the model to correct for potential standard error bias. The results (see Supplementary Materials Table S1, Model 3) showed that after accounting for cluster effects and adjusting the set of control variables, the estimated results of the core variables remained stable, which further validates the robustness of the main findings in this study.

#### 3.5.2. Replacing the Benchmark Regression Model

Since the dependent variable (internet addiction) in this study is constructed as the sum of dichotomous items, with a total score ranging from 0 to 8, it represents bounded data. The mediation model used in this study is essentially based on OLS regression. Although OLS regression can produce consistent estimates under large sample conditions even if the dependent variable is bounded, potential bias may still arise due to the scale properties of the dependent variable. Therefore, we classified participants with an internet addiction score ≥ 5 as having internet addiction and those with a score < 5 as not having internet addiction. Using this ≥5 cutoff, the prevalence rate of internet addiction in the current sample was 15.44%. This threshold was selected based on prior validation studies of Young’s internet addiction questionnaire in Chinese adolescent populations, which has been widely adopted in peer research and demonstrated good discriminant validity for identifying clinically relevant internet addiction risk [44]. We then performed binary logistic regression analysis. Control variables included age, gender, household registration type, residence type, ethnicity, only-child status, father’s education, mother’s education, and parental relationship. As shown in Supplementary Materials Table S2, the estimates for the core variables remained consistent, which further supports the credibility of our key conclusions.

## 4. Discussion

This study systematically examined the mechanisms underlying the relationship between emotional eating behavior and internet addiction among 3245 junior high school students in Shenzhen, China, and tested the mediating roles of sleep quality (PSQI scores, with higher scores indicating worse sleep quality) and depression. The findings supported all hypotheses, offering novel insights into the developmental pathways of adolescent internet addiction.

First, emotional eating behavior was found to directly positively associated with internet dependence in adolescents. This aligns with the behavioral mechanisms of emotional eating: although consuming highly palatable foods may temporarily alleviate stress and anxiety, emotional eating is fundamentally a maladaptive emotion regulation strategy [45]. Such behavior fails to resolve negative emotions effectively and may instead generate new stressors, which is linked to individuals to seek compensatory relief in cyberspace. Prior research suggests the immediate feedback from gaming achievements and social interactions in online environments shares dopaminergic reward circuitry with emotional eating, which may provide a theoretical context for the observed association between emotional eating and reinforced dependence on internet use [46]. Notably, although this direct association was statistically significant, the effect size was small in magnitude, indicating that emotional eating accounts for only a modest proportion of variance in internet addiction. This suggests that emotional eating may function as one incremental risk indicator among multiple psychosocial factors for adolescent internet addiction, rather than as a strong standalone predictor.

Second, sleep quality (PSQI scores, with higher scores indicating worse sleep quality) partially mediated the relationship between emotional eating and internet addiction, consistent with previous findings that poor sleep is associated with internet addiction [47]. This finding aligns with research by Jawarneh [48], who reported that unhealthy eating behaviors, particularly consuming heavy evening meals and substituting snacks for main meals, were significantly associated with poor sleep quality. These results suggest that the specific dietary patterns characterizing emotional eating may directly contribute to sleep disturbances. From a physiological perspective, prior literature indicates emotional eating often involves high-sugar and high-fat food consumption, which may be linked to disrupted sleep–wake cycle by activating the orexin system [49]. As documented in previous studies, sleep deprivation is associated with sensitivity in the dopaminergic reward system, lowering the threshold for craving online stimuli, and higher levels of uncontrolled internet use [46]. Furthermore, the relationship between sleep and internet use appears bidirectional. Thottan et al. [50] demonstrated that severe internet addiction was associated with 3.10 times higher odds of not meeting sleep guidelines, while moderate and severe internet addiction were associated with later bedtimes. Although their main analyses did not find significant associations with sleep quality, sensitivity analyses suggested potential links, indicating that the relationship between internet addiction and sleep outcomes may be complex and context-dependent.

Critically, the mediating role of sleep quality (PSQI scores, with higher scores indicating worse sleep quality) suggests that emotional eating is associated with internet addiction partly through pathways related to sleep disturbance, which prior research has linked to circadian rhythm dysregulation, highlighting a potential theoretical target for early intervention.

Furthermore, depression also exhibited a partial mediating effect between emotional eating and internet addiction. As a core symptom of atypical depression, the association between emotional eating and depressive symptoms has been validated in large-scale epidemiological studies [51,52]. This study extends these findings by demonstrating that depressive states are associated with individuals escaping real-world distress through internet use, mechanistically echoing Ye et al.’s meta-analytic conclusion (based on over 100,000 samples) that depression is significantly associated with adolescent internet addiction risk [53]. This pattern is consistent with large-scale epidemiological evidence from the same geographic region. Pan et al. [54], in a study of 67,182 adolescents from Shenzhen, found that internet addiction exhibited significant associations with increased risks of depression across early, middle, and late adolescence. Notably, their study also revealed distinct association patterns between internet addiction and sleep disturbance across different developmental stages, underscoring the complex interplay among these variables. Importantly, the mediating role of depression underscores the psychopathological mechanisms underlying emotional eating: when food intake fails as an emotion regulation strategy, accumulated depressive emotions are associated with impaired self-control capacities, which is linked to individuals adopting internet use as a new emotional outlet.

The present study identified a statistically significant chain-mediation pattern linking emotional eating, sleep quality, depression, and internet addiction within cross-sectional data. It should be explicitly clarified that these analyses reflect statistical mediation associations, which are consistent with—but do not establish—the proposed temporal or developmental pathway of “emotional eating → sleep quality → depression → internet addiction” (sleep quality was measured by PSQI, with higher scores indicating worse sleep quality). Specifically, the observed statistical associations are consistent with a pathway wherein emotional eating may first be linked to sleep disturbances, which prior theoretical frameworks suggest may be associated with disrupted homeostatic balance of the brain’s reward system. This subsequently triggers depressive emotions at the psychological level, reducing tolerance for negative affect and ultimately higher levels of dysregulated internet use. This observed statistical mediation pattern is consistent with a model wherein the association between emotional eating and internet addiction may be shaped by the interplay of physiological and psychological factors, which may provide a theoretical basis for exploring interventions targeting the examined variables in future longitudinal research. It should be noted that the cross-sectional design of this study cannot confirm that targeting any single component would disrupt risk transmission in a developmental context. This integrated perspective is supported by research examining similar mechanisms. Helin et al. [55] investigated the relationships among emotion dysregulation, internet addiction, and eating behaviors in adolescents, finding that the association between emotion dysregulation and body mass index was totally mediated by internet addiction and uncontrolled/emotional eating. Their study further revealed a 22-fold increased risk of obesity in adolescents with moderate/severe internet addiction compared to those without addiction. These findings parallel our results, suggesting that internet addiction and maladaptive eating behaviors frequently co-occur and share common underlying mechanisms related to emotion regulation difficulties. However, while Helin et al. [55] focused on obesity as the outcome, the present study extends this line of inquiry by identifying the specific chain of mechanisms linking emotional eating to internet addiction through sleep quality and depression in a non-clinical junior high school population.

The innovations and strengths of the present study are summarized as follows. First, to our knowledge, few studies have developed an integrated multiple mediation model examining the association between emotional eating behavior and internet addiction among Chinese junior high school students, and identified a statistical chain-mediation pattern linking emotional eating, sleep quality, depression, and internet addiction, which extends beyond traditional single-factor correlation and single-mediator analyses. Second, the current study integrates behavioral, physiological, and psychological mechanisms into a unified analytical framework, providing empirical evidence for the multilevel “behavior–physiology–psychology” associative framework. Third, this study is based on a large sample of well-defined adolescent students at school, which greatly improves the reliability and representativeness of the findings.

In terms of practical implications, given the small effect sizes observed and the modest proportion of variance in internet addiction explained by emotional eating, the findings support the inclusion of emotional regulation and sleep hygiene within broader, multi-component prevention or intervention frameworks for adolescent internet addiction, rather than supporting interventions targeting emotional eating alone as a standalone strategy to substantially reduce risk. Specifically, schools and families may establish a tiered monitoring and support system: for students exhibiting emotional eating behavior, sleep quality (e.g., difficulty falling asleep, frequency of nocturnal awakenings) and depressive symptoms (e.g., decreased interest, self-denial) should be monitored simultaneously; for individuals with poor sleep quality, sleep hygiene education and cognitive behavioral therapy for insomnia can be provided; for those who have developed depressive tendencies, standardized psychological counseling and standardized internet use guidance should be combined to address the co-occurring risk factors for internet addiction.

This study has several limitations that should be acknowledged when interpreting the findings: (a) This study adopted a cross-sectional design, which cannot clarify the causal temporal relationship between variables. Consistent with this limitation, possible bidirectionality among key variables (including sleep quality and internet use, depressive symptoms and internet use, emotional eating and mediating variables) cannot be ruled out, even though the results are aligned with our proposed theoretical model. Longitudinal or experimental designs are required in future research to verify the causal direction and developmental pathways between variables. (b) The effect sizes observed in this study were small in magnitude. Specifically, the correlations between emotional eating behavior and core study variables (internet addiction, sleep quality, depressive symptoms) were statistically significant but weak (r ranged from 0.066 to 0.113), and the mediating effects accounted for a relatively modest proportion of the total effect. While the small effect sizes suggest that emotional eating alone has limited utility as a standalone predictor of internet addiction, the significant mediating pathways identified in this study provide valuable theoretical insights into the process by which it may exert its influence. (c) This study relied exclusively on self-report measures for all variables, which may introduce response biases such as social desirability bias. In addition, all data were collected at a single time point within the same classroom setting, creating a risk of shared method variance. Although Harman’s single-factor test did not detect significant common method bias, this method has inherent limitations, and the potential for common method variance cannot be completely eliminated, which may have a subtle impact on the stability of the results. (d) There are inherent constraints associated with modeling a bounded outcome variable. The dependent variable (internet addiction score) was constructed as the sum of dichotomous items, with a fixed range of 0 to 8. Although the sensitivity analysis using binary logistic regression confirmed the robustness of the core findings, the bounded nature of the score may still introduce potential biases in the OLS regression-based mediation model, limiting the precision of effect estimates. (e) All participants were recruited from a single city in China, so the generalizability of the findings to other regions, age groups, or populations needs to be further verified in multi-regional and multi-sample studies. Future research will address the above limitations to improve the robustness and generalizability of the findings.

## 5. Conclusions

This study confirms that emotional eating behavior in junior high school students is positively associated with internet addiction through both independent and chain-mediated effects of sleep quality (PSQI scores, with higher scores indicating worse sleep quality) and depression. The findings are consistent with a statistical mediation pattern linking emotional eating, sleep quality, depressive symptoms, and internet addiction, corresponding to the sequence of “maladaptive emotion regulation → circadian disruption → psychopathology → addictive behavior”; however, longitudinal research is needed to determine whether this sequence reflects a definitive developmental or risk transmission pathway. Based on these findings, future studies should employ longitudinal designs to establish causal relationships among these variables and examine potential bidirectional effects, particularly whether internet addiction may in turn exacerbate emotional eating behaviors. Additionally, intervention studies targeting sleep quality improvement and emotional regulation training could test whether modifying these mediators effectively reduces internet addiction risk. Future research should also investigate whether these mechanisms generalize across different developmental stages, cultural contexts, and clinical populations, and explore whether similar statistical mediation patterns exist for other addictive behaviors or mental health outcomes in adolescents.

## Figures and Tables

**Table 1:** Differences in Internet Addiction among Junior High School Students across Different Demographic Characteristics.

Variables	Variable Assignment	M (P_25_, P_75_)/ N (Percentage)	Internet Addiction		
			*Z*	*H*	*p*
Age	Continuous type	13.90 (13.20, 14.60)	—		—
Gender	1 = Male	1734 (53.44%)	−2.546 *	—	0.011
	2 = Female	1511 (46.56%)			
Hukou	0 = Non-local Hukou	1985 (61.17%)	−1.427	—	0.153
	1 = Local Hukou	1260 (38.83%)			
Residential area	1 = Community	2506 (77.23%)	—	1.387	0.500
	2 = Urban village	710 (21.88%)			
	3 = Other	29 (0.89%)			
Ethnicity	1 = Han	3102 (95.59%)	−1.909	—	0.056
	2 = Ethnic minority	143 (4.41%)			
Only child status	1 = Yes	482 (14.85%)	−0.883	—	0.377
	2 = No	2763 (85.15%)			
Father’s education level	1 = No schooling	3 (0.09%)	—	5.394	0.612
	2 = Primary school	129 (3.98%)			
	3 = Junior high school	901 (27.77%)			
	4 = Vocational high school	413 (12.73%)			
	5 = Senior high school	525 (16.18%)			
	6 = Junior college	516 (15.90%)			
	7 = Bachelor’s degree	623 (19.20%)			
	8 = Master’s degree or above	135 (4.16%)			
Mother’s education level	1 = No schooling	17 (0.52%)	—	13.639	0.058
	2 = Primary school	216 (6.66%)			
	3 = Junior high school	982 (30.26%)			
	4 = Vocational high school	359 (11.06%)			
	5 = Senior high school	507 (15.62%)			
	6 = Junior college	517 (15.93%)			
	7 = Bachelor’s degree	566 (17.44%)			
	8 = Master’s degree or above	81 (2.50%)			
Parental relationship status	1 = Very poor	66 (2.03%)	—	118.924 **	<0.001
	2 = Relatively poor	136 (4.19%)			
	3 = Average	578 (17.81%)			
	4 = Relatively good	1222 (37.66%)			
	5 = Very good	1243 (38.31%)			

Note: * *p* < 0.050; ** *p* < 0.010; *Z* represents the Mann–Whitney *U* test; *H* represents the Kruskal–Wallis *H* test.

**Table 2:** Descriptive statistics and correlations between the main variables.

Variables	M	SD	EEB	IA	SQ	Dn
EEB	12.272	4.779	1.000			
IA	1.864	2.351	0.066 **	1.000		
SQ	4.982	3.003	0.092 **	0.342 **	1.000	
Dn	7.126	8.717	0.113 **	0.365 **	0.510 **	1.000

** *p* < 0.01; EEB = Emotional Eating Behavior; IA = Internet Addiction; SQ = Sleep Quality Index; Dn = Depression.

**Table 3:** Mediation analysis of chain-mediation models.

Model Pathways	*β*	*SE*	95%CI		Mediating Effect (%)
			LLCI	ULCI	
Total effect	0.0427	0.0085	0.0261	0.0594	100.00
Direct effect					
EEB → IA	0.0240	0.0081	0.0082	0.0399	56.21
Indirect effect					
EEB → IA	0.0187	0.0032	0.0126	0.0252	43.79
EEB → SQ → IA	0.0062	0.0017	0.0033	0.0098	14.52
EEB → Dn → IA	0.0084	0.0020	0.0047	0.0125	19.67
EEB → SQ → Dn → IA	0.0041	0.0010	0.0022	0.0062	9.60

EEB = Emotional Eating Behavior; IA = Internet Addiction; SQ = Sleep Quality Index; Dn = Depression.

## Data Availability

The datasets produced and analyzed in the present study are not publicly accessible at this time due to concurrent related research initiatives. However, they can be made available by the corresponding author upon receiving a justifiable request.

## Supplementary Materials

The following supporting information can be downloaded at: https://www.mdpi.com/article/10.3390/nu18050800/s1, Table S1: Results of the baseline regression (OLS); Table S2: Regression Results with a Binary Dependent Variable.
