# Supplementary material for: The Relationship Between Emotional Eating Behavior and Internet Addiction in Junior High School Students: A Cross-Sectional Study
Source: Nutrients. 2026 Feb 28;18(5):800. doi: 10.3390/nu18050800 (PMC12986662; doi:10.3390/nu18050800)
Supplement: Supplementary file 1 [file nutrients-18-00800-s001.zip › nutrients-4138804-supplementary.pdf]

**Supplementary Material**

**Table S1** Results of the baseline regression (OLS).

**Table S2** Regression Results with a Binary Dependent Variable.

**Table S1** Results of the baseline regression (OLS).

| Variables | Variable assignment | Model 1 | | Model 2 | | Model 3 | |
|---|---|---|---|---|---|---|---|
| | | Coefficient | Standard Error | Coefficient | Standard Error | Coefficient | Standard Error |
| Emotional eating behavior | Continuous type | 0.042** | 0.009 | 0.042** | 0.009 | 0.042** | 0.007 |
| Age | Continuous type | | | 0.044 | 0.046 | 0.044 | 0.102 |
| Gender | 1=Male | | | | | | |
| | 2=Female | | | 0.009 | 0.082 | 0.009 | 0.076 |
| Hukou | 0=Non-local Hukou | | | | | | |
| | 1=Local Hukou | | | 0.118 | 0.097 | 0.118 | 0.096 |
| Residential area | 1=Community | | | | | | |
| | 2=Urban village | | | 0.101 | 0.100 | 0.101 | 0.073 |
| | 3=Other | | | -0.026 | 0.434 | -0.026 | 0.381 |
| Ethnicity | 1=Han | | | | | | |
| | 2=Ethnic minority | | | 0.337 | 0.199 | 0.337* | 0.134 |
| Only child status | 1=Yes | | | | | | |
| | 2=No | | | 0.101 | 0.117 | 0.101 | 0.086 |
| Father's education level | 1=No schooling | | | | | | |
| | 2=Primary school | | | 1.518 | 1.408 | 1.518** | 0.447 |
| | 3=Junior high school | | | 1.637 | 1.399 | 1.637** | 0.481 |
| | 4=Vocational high school | | | 1.623 | 1.403 | 1.623** | 0.441 |
| | 5=Senior high school | | | 1.668 | 1.401 | 1.668** | 0.473 |
| | 6=Junior college | | | 1.701 | 1.403 | 1.701** | 0.506 |
| | 7=Bachelor's degree | | | 1.674 | 1.403 | 1.674** | 0.516 |
| | 8=Master's degree or above | | | 1.491 | 1.420 | 1.491* | 0.573 |
| Mother's education level | 1=No schooling | | | | | | |
| | 2=Primary school | | | 0.397 | 0.605 | 0.397 | 0.386 |
| | 3=Junior high school | | | 0.076 | 0.589 | 0.076 | 0.350 |

| | | | | | | | | |
|---|---|---|---|---|---|---|---|---|
| | 4=Vocational high school | | | 0.442 | 0.601 | 0.442 | 0.395 |
| | 5=Senior high school | | | 0.009 | 0.596 | 0.009 | 0.359 |
| | 6=Junior college | | | 0.061 | 0.598 | 0.061 | 0.427 |
| | 7=Bachelor's degree | | | 0.086 | 0.601 | 0.086 | 0.372 |
| | 8=Master's degree or above | | | 0.025 | 0.669 | 0.025 | 0.347 |
| Parents' Relationship Quality | 1=Very poor | | | | | | |
| | 2=Relatively poor | | | 0.351 | 0.348 | 0.351 | 0.388 |
| | 3=Average | | | -0.008 | 0.302 | -0.008 | 0.332 |
| | 4=Relatively good | | | -0.446 | 0.294 | -0.446 | 0.268 |
| | 5=Very good | | | -1.027** | 0.294 | -1.027** | 0.292 |
| Constant Term | | -0.667 | 1.510 | -0.667 | 1.510 | -0.667 | 1.688 |
| Control Variables | | No | | Yes | | Yes | |
| School-level controls | | No | | No | | Yes | |
| Sample Size | | 3245 | | 3245 | | 3245 | |
| $R^2$ | | 0.047 | | 0.047 | | 0.047 | |

Note: * $P$<0.050; ** $P$<0.010.

**Table S2** Regression Results with a Binary Dependent Variable.

| Variable | | with internet addiction[a] | | | |
|---|---|---|---|---|---|
| | | B | Standard Error | Wald | OR |
| Emotional eating behavior | Continuous type | 0.041** | 0.011 | 14.701 | 1.042 |
| Sleep quality | Continuous type | 0.081** | 0.019 | 18.220 | 1.085 |
| Depression | Continuous type | 0.060** | 0.006 | 102.832 | 1.062 |
| Age | Continuous type | -0.082 | 0.060 | 1.886 | 0.921 |
| Gender | 1=Male | 0.282** | 0.107 | 6.957 | 1.326 |
| | 2=Female | 0[b] | | | |
| Hukou | 0=Non-local Hukou | 0.051 | 0.124 | 0.167 | 1.052 |
| | 1=Local Hukou | 0[b] | | | |
| Residential area | 1=Community | -0.216 | 0.564 | 0.146 | 0.806 |
| | 2=Urban village | -0.016 | 0.570 | 0.001 | 0.984 |
| | 3=Other | 0[b] | | | |
| Ethnicity | 1=Han | -0.480* | 0.227 | 4.459 | 0.619 |
| | 2=Ethnic minority | 0[b] | . | . | . |
| Only child status | 1=Yes | -0.064 | 0.152 | 0.178 | 0.938 |
| | 2=No | 0[b] | | | |
| Father's education level | 1=No schooling | -17.828 | 0.000 | | 1.808E-8 |
| | 2=Primary school | -0.092 | 0.420 | 0.048 | 0.912 |
| | 3=Junior high school | -0.107 | 0.345 | 0.097 | 0.898 |
| | 4=Vocational high school | -0.079 | 0.354 | 0.050 | 0.924 |
| | 5=Senior high school | -0.081 | 0.351 | 0.053 | 0.922 |
| | 6=Junior college | 0.038 | 0.342 | 0.012 | 1.038 |
| | 7=Bachelor's degree | 0.064 | 0.331 | 0.038 | 1.067 |
| | 8=Master's degree or above | 0[b] | | | |
| Mother's education level | 1=No schooling | -0.607 | 0.927 | 0.428 | 0.545 |
| | 2=Primary school | 0.381 | 0.463 | 0.677 | 1.464 |
| | 3=Junior high school | 0.126 | 0.434 | 0.084 | 1.134 |
| | 4=Vocational high school | 0.383 | 0.442 | 0.751 | 1.467 |
| | 5=Senior high school | 0.128 | 0.439 | 0.086 | 1.137 |
| | 6=Junior college | -0.002 | 0.432 | 0.000 | 0.998 |
| | 7=Bachelor's degree | 0.143 | 0.424 | 0.114 | 1.154 |
| | 8=Master's degree or above | 0[b] | | | |
| Parents' Relationship Quality | 1=Very poor | 0.180 | 0.359 | 0.252 | 1.198 |
| | 2=Relatively poor | 0.548* | 0.241 | 5.185 | 1.731 |
| | 3=Average | 0.705** | 0.149 | 22.482 | 2.024 |
| | 4=Relatively good | 0.403** | 0.130 | 9.691 | 1.497 |

| | | | | |
|---|---|---|---|---|
| | 5=Very good | $0^b$ | | |
| Intercept | | -2.072 | 1.098 | 3.561 |

Note: * *P*<0.050; ** *P*<0.010; a: comparison with the "no internet addiction" category; b: this parameter is set to zero because it is redundant.
